# Synteny conservation between two distantly-related Rosaceae genomes: *Prunus *(the stone fruits) and *Fragaria *(the strawberry)

**DOI:** 10.1186/1471-2229-8-67

**Published:** 2008-06-18

**Authors:** Santiago Vilanova, Daniel J Sargent, Pere Arús, Amparo Monfort

**Affiliations:** 1 IRTA. Centre de Recerca en Agrigenòmica CSIC-IRTA-UAB, 08348 Cabrils, Spain; 2 East Malling Research (EMR), East Malling, Kent, ME19 6BJ, UK; 3 Universidad Politécnica de Valencia, Centro de Conservación y Mejora de la Agrodiversidad Valenciana (COMAV), Spain

## Abstract

**Background:**

The Rosaceae encompass a large number of economically-important diploid and polyploid fruit and ornamental species in many different genera. The basic chromosome numbers of these genera are *x *= 7, 8 and 9 and all have compact and relatively similar genome sizes. Comparative mapping between distantly-related genera has been performed to a limited extent in the Rosaceae including a comparison between *Malus *(subfamily Maloideae) and *Prunus *(subfamily Prunoideae); however no data has been published to date comparing *Malus *or *Prunus *to a member of the subfamily Rosoideae. In this paper we compare the genome of *Fragaria*, a member of the Rosoideae, to *Prunus*, a member of the Prunoideae.

**Results:**

The diploid genomes of *Prunus *(*2n *= *2x *= 16) and *Fragaria *(*2n *= *2x *= 14) were compared through the mapping of 71 anchor markers – 40 restriction fragment length polymorphisms (RFLPs), 29 indels or single nucleotide polymorphisms (SNPs) derived from expressed sequence tags (ESTs) and two simple-sequence repeats (SSRs) – on the reference maps of both genera. These markers provided good coverage of the *Prunus *(78%) and *Fragaria *(78%) genomes, with maximum gaps and average densities of 22 cM and 7.3 cM/marker in *Prunus *and 32 cM and 8.0 cM/marker in *Fragaria*.

**Conclusion:**

Our results indicate a clear pattern of synteny, with most markers of each chromosome of one of these species mapping to one or two chromosomes of the other. A large number of rearrangements (36), most of which produced by inversions (27) and the rest (9) by translocations or fission/fusion events could also be inferred. We have provided the first framework for the comparison of the position of genes or DNA sequences of these two economically valuable and yet distantly-related genera of the Rosaceae.

## Background

Genome comparisons based on the map position of homologous markers between different plant taxa have established that the genomes of species within families of the Plant Kingdom, such as Solanaceae [1,2], Poaceae [3], Fabaceae [4] and Brassicaceae [5] differ in a limited number of chromosomal rearrangements, meaning that extensive chromosomal regions, and even entire chromosomes, are syntenic and colinear between different species [6]. However, conservation of synteny is greatly reduced when comparisons are made between members of different families, as shown through the comparison of the genomes of several crop species with that of *Arabidopsis thaliana *[7-9].

The Rosaceae encompass a large number (3,000) of diploid and polyploid species [10] including important crops, such as those belonging to the genera *Prunus *(almond and all stone fruits: peach, apricot, cherry and plum), *Malus *(apple), *Pyrus *(pear), *Rosa *(rose), *Rubus *(raspberry) and *Fragaria *(strawberry). Their basic chromosome numbers are *x *= 7, 8 and 9 and all have a compact and relatively similar genome size that for diploid species ranges from ~170 Mbp in *Fragaria *(2n = 2x = 14) [11] to ~300 Mbp in *Prunus *(2*n *= 2*x *= 16) [12]. Most of the polyploid species within the family have a genome size approximately proportional to that of the diploid genomes from which they are composed, i.e. the amphidiploid apple (2*n *= 2*x *= 34) has a genome size of ~750 Mbp [12] and the cultivated strawberry, an allo-octoploid (2*n *= 8*x *= 56), has a genome size of ~800 Mbp [11].

Comparative mapping has been performed to a limited extent in the Rosaceae [13]. The genus *Prunus*, from the subfamily Prunoideae, was the first to be studied, due to the existence of a high density reference map [14,15], based on a highly polymorphic interspecific (almond × peach) F_2 _population and constructed with markers transferable to other species within the genus (mainly RFLPs and SSRs). The analysis of 16 published maps of *Prunus *species, each with at least 28 markers common to the reference map, established that the *Prunus *genome is essentially colinear and shared by all diploid species studied so far (peach, almond, apricot, cherry, myrobolan plum, *P. davidiana*, and *P. ferganenesis*) [13]. Comparisons between the genomes of apple and pear, which belong to the subfamily Maloideae, indicate a very high level of synteny, and no major chromosomal rearrangements can be deduced from studies of common markers mapped in these genera [16,17]. No data are currently published on synteny studies between members of the subfamily Rosoideae, which includes strawberry, rose and raspberry. There is however a partial comparison between the genomes of genera belonging to different sub-families: *Prunus *(Prunoideae) and *Malus *(Maloideae). This work was done with 34 markers (23 RFLPs and 11 isoenzymes) and, where comparisons were possible, a high level of synteny was detected, as well as at least one large-scale chromosomal rearrangement [14].

In this paper we have compared the genomes of *Prunus *and *Fragaria*. For this purpose, we have used the previously mentioned *Prunus *reference map and the *Fragaria *reference map [18], which is based on an interspecific diploid *F. vesca *× *F. nubicola *F_2 _population. The *Fragaria *map was constructed with mainly SSRs and markers derived from the growing information existing on expressed sequence tag (EST) sequences of different Rosaceae species, which are available through the Genome Database for the Rosaceae [19]. Here, we have established a framework with common markers covering both genomes and found that in spite of the conservation of large chromosomal fragments, as expected from confamilial species, many chromosomal rearrangements separate the diploid *Fragaria *and *Prunus *genomes, supporting their distant position within the family deduced from DNA sequence data [20].

## Results

### *Fragaria *and *Prunus maps*

In total, 71 anchor markers were available for the comparison between the diploid genomes of *Prunus *and *Fragaria*. These were: a) 40 RFLPs from the same number of probes found polymorphic out of 65 single-copy probes already mapped in *Prunus *(Table 1), b) 13 *Fragaria *ESTs obtained after selecting from 135 of them highly homologous to *Prunus *mapped ESTs out of the 515 ESTs of the *Prunus *transcript map (Table 2); these markers were selected by their position on the map as they fell in regions not covered with RFLPs or that were judged to be of interest for the genome comparison, c) eight *Prunus *ESTs selected from 13 ESTs of the transcript map that did not have sequence homology with known *Fragaria *ESTs (Table 2), d) eight *Fragaria *STSs obtained from genes of known function previously mapped in *Fragaria *[21] (Table 2), and e) two SSRs (Table 2).

**Table 1:** RFLP probes mapped in *Prunus *and *Fragaria *Single-copy RFLP probes mapped in *Prunus *and used for mapping in *Fragaria*. RFLP position in the *Prunus *and *Fragaria *maps and estimated copy number in *Fragaria*

		*Prunus*		*Fragaria*		
			
Marker	Accession No.	LG^a^	cM	LG^a^	cM	Copy no.
**AG53**	BH023829	PG1	2.5	FG4	22.1	2
**AC24**	BI203138	PG1	4.3	FG4	20.3	1
**AG102**	BH023845	PG1	8.7	FG6	63.4	2
**PC78**	BI203148	PG1	13.6	FG4	31.1	1
**AC32**	BI203135	PG1	25.8	FG4	0	2
**FG16**	BH023876	PG1	37	+^c^	-	-
**PC85**	n.d.^b^	PG1	37	+	-	-
**PC15**	BI203140	PG1	43	+	-	-
**AG47**	BH023861	PG1	43.7	FG2	17.4	2
**PC7**	n.d.	PG1	44	++^d^	-	-
**FG36**	BH023883	PG1	65.1	FG2	36.8	1
**AC13**	BI203106	PG2	6	+	-	-
**AC31**	BI203096	PG2	7.9	FG7	0	1
**AC10**	BI203087	PG2	8.1	FG4	13.5	2
**PC5**	BI203107	PG2	21	+	-	-
**AG35**	BH023896	PG2	25	FG7	9	1
**AC19**	BI203097	PG2	37	++	-	-
**MC045**	BI203117	PG2	38	FG7	81	2
**Ole1**	X78118	PG2	39	+	-	-
**Omt1**	X83217	PG2	47.6	FG7	89.6	1
**CC125**	n.d.	PG2	49	+	-	-
**MC115**	n.d.	PG3	0	++	-	-
**FG13**	n.d.	PG3	6	++	-	-
**AG56**	n.d.	PG3	6.4	FG6	65.2	1
**AG7**	BH023839	PG3	12	+	-	-
**CC116**	n.d.	PG3	22	FG6	4.7	1
**CC2**	BI203070	PG3	27.7	FG6	43.7	2
**CC8**	BI203091	PG3	34.4	FG6	81.9	1
**AG106**	BH023814	PG3	37.1	FG6	96	2
**CC47**	BI203119	PG4	5.4	FG3	7.3	1
**AG6**	BH023828	PG4	24.1	FG3	42.2	1
**FG3**	BH023837	PG4	37.7	FG3	36.5	1
**PC1**	BI203133	PG4	49.9	FG3	48.2	1
**PLG35**	n.d.	PG5	0	m^e^	-	-
**AC49**	BI203072	PG5	15.2	FG5	0	1
**PC14**	BI203132	PG5	20.6	FG5	19.1	1
**AG33**	BH023821	PG5	49.1	FG5	56.4	1
**AG13**	n.d.	PG6	3	+	-	-
**AG54**	n.d.	PG6	3	+	-	-
**AG40**	BH023810	PG6	5	+	-	-
**PLG59**	n.d.	PG6	5	+	-	-
**FG215**	BH023827	PG6	8.7	FG3	36.5	1
**AC50**	BI203094	PG6	17.5	FG3	48.2	2
**AC8**	BI203052	PG6	34.5	FG1	61.2	2
**PC21**	n.d.	PG6	56.4	FG1	72.8	2
**PC73**	n.d.	PG6	64	m	-	-
**PC60**	n.d.	PG6	70	+	-	-
**Pgl1**	X75020	PG6	74.3	FG6	22.8	1
**LTP2**	n.d.	PG6	78	+	-	-
**AC44**	BI203095	PG7	10.3	FG2	77.1	2
**PC12**	n.d.	PG7	24.7	FG6	102.7	1
**MC225**	BI203059	PG7	28.4	FG6	61.5	1
**AG104**	BH023923	PG7	31.2	FG6	57	1
**TSA3**	n.d.	PG7	52.9	FG1	39.3	1
**FG42**	n.d.	PG7	59	m	-	-
**FG27**	BH023875	PG7	63	+	-	-
**CC132**	BI203120	PG7	67.6	FG3	38.3	2
**FG24**	BH023858	PG7	80	++	-	-
**EXT1**	n.d.	PG8	4	++	-	-
**LY29**	BH023873	PG8	20.8	FG2	19.5	2
**PC101**	BI203099	PG8	29.3	FG2	19.7	1
**FG37**	BH023891	PG8	40.9	FG2	25.3	1
**AG49**	BH023870	PG8	49.1	FG6	61.5	1
**AC26**	BI203074	PG8	52	++	-	-
**Pru1**	X78119	PG8	53.1	FG2	26.7	1
**PC36**	n.d.	PG8	60	+	-	-

^a^LG = Linkage group in Fragaria (F) and Prunus (P)

^b^n.d. = not determined

^c^+ = no or weak hybridization

^d^++ = complex banding pattern

^e^m = monomorphic

**Table 2:** Gene and EST-based markers mapped in *Prunus *and *Fragaria*.

	*Prunus *			*Fragaria *			TBLASTX		
			
Locus	Accession number	LG^a^	cM^b^	Accession number	LG^a^	cM^b^	E-value^c^	Swissprot-homology^d^	Marker^e^
EFvVB2119	BU048565	PG1	4.3	CX662119	FG6	22.5–43.2	5,00E-49	small GTP binding protein	SNP
EFvVB2179	BU046414	PG1	4.3	CX662179	FG4	0.0–26.0	2,00E-78	Aldoketo-reductase	SNP
EFvNH8894	BU042720	PG1	40.5	DV438894	FG2	53.9–73.8	3.00E-84	auxin-induced protein (Aux22)	SNP
EFaUF6868	BU046817	PG1	48.0	CO816868	FG2	53.9–73.8	1.00E-109	20S proteasome alpha 6 subunit	Indel
EFaTR1976	BU039761	PG1	48.6	CO381976	FG2	53.9–73.8	2.00E-88	Mannan endo-1.4-Beta-Mannosidase	SNP
EKO	AF495728	PG1	35.7–49.8	AY462247	FG2	28.9	8.00E-155	Ent-kaurene oxidase	Indel
DFR	AB095030	PG1	35.7–49.8	AY575057	FG2	70.5	2.00E-106	dihydroflavonol reductase	Indel
EFvVB1231	BU046687	PG1	41.3	CX661231	FG4	26.0–46.1	5.00E-17	RAD23-like	SNP
EPpCU7308	BU047308	PG1	75.2	-	FG2	26.7–45.8	5.00E-27	electron carrier/iron ion binding	Indel
EPpCU9642	BU039642	PG1	40.5	-	FG2	0.0–26.7	4.00E-72	ACT domain-containing protein	Indel
EFaUF7699	BU040484	PG2	7.9	CO817699	FG4	0.0–26.0	1.00E-102	Luminal Binding Protein BiP	Indel
EFaUF7084	BU046792	PG2	24.3	CO817084	FG7	63.4–81.0	3.00E-96	40 S ribosomal protein	SNP
EPpCU2875	BU042875	PG2	25.0	-	FG7	20.3	4.00E-132	RNA helicase	Indel
EFvNH8484	BU041902	PG2	39.4	DV438484	FG7	27.0–38.6	5.00E-105	GTP-Binding protein	SNP
EPpCU9223	BU039223	PG2	39.4	DY672045	FG7	44.5	1.00E-48	6-phosphofructokinase	Indel
ACO	AF129073	PG3	35.0	AY706156	FG6	83.1	1.00E-165	ACC oxydase	SSR
EFvVB2013	BU039972	PG5	0.0	CX662013	FG5	50.4–72.5	6.00E-83	Pectinacetylesterase precursor	Indel
ANS	AB097216	PG5	15.2–21.0	AY695818	FG5	9.7	4.00E-143	Anthocyanidin shynthase	Indel
CEL-2	AJ890498	PG5	21.7–40.7	AF054615	FG5	29.2	0.0	endo-beta-1,4-glucanase	Indel
AMPA112	AY377916	PG5	4.1	-	FG5	64.5	-	--	SSR
EFvNH9852	BU040757	PG6	6.4	DV439852	FG7	27.0–38.6	1.00E-137	60S Ribosomal Protein L10	SNP
EPpCU9257	BU039257	PG6	17.5	-	FG7	24.9	7.00E-109	phosphoglucomutase precursor	Indel
EPpCU1785	BU041785	PG6	79.6	DY669394	FG1	45.2–47.9	1.00E-12	SNF4 (Sucrose NonFermenting 4)	SNP
EPpCU1830	BU041830	PG6	79.6	CX661290	FG6	14.1	1.00E-132	26s proteasome aaa-atpase subunit rpt5a	Indel
APX	EE488129	PG6	4.1–24.9	AF158654	FG3	49.5	4.00E-102	L-ascorbate peroxidase	Indel
EFaUF7248	BU043308	PG7	10.3	CO817248	FG2	53.9–73.8	1.00E-97	Methionine synthase	Indel
EFvVB1923	BU039764	PG7	29.6	CX661923	FG6	56.5–68.1	2.00E-73	Enolase	SNP
EPpCU9910	BU039910	PG7	64.7	-	FG1	8.3	1.00E-62	putative ethanolamine kinase 1	Indel
F3H	AB097151	PG7	42.5–47.8	AB201760	FG1	40.6	2.00E-178	flavanone 3-hydroxylase	Indel
PES	X95991	PG7	49–56.1	AY324809	FG1	33.7	0.0	Pectinesterase	Indel
ADH	BU573880	PG8	0.0–10.9	X15588	FG2	17.9	4.00E-101	alcohol dehydrogenase	SNP

Gene or EST-based and SSR markers mapped in the *Prunus *and *Fragaria *genomes, with their accession numbers, map positions and homology with known proteins of other species.

^a ^LG = linkage group

^b ^Map position: If the marker was bin-mapped, the interval covered by the bin where the marker is located

^c ^E-value at NCBI database restricted to plant sequences

^d ^Predicted function

^e ^Marker type detected

Two mapping strategies were used in the placement of novel markers on both the T×E and FV×FN linkage maps (Table 3). For 55 markers: all 40 RFLPs, the eight *Fragaria *STSs, five of the *Fragaria *ESTs that displayed intron length polymorphisms and the two SSRs, were mapped by genotyping all individuals of the FV×FN population. The 16 remaining markers were mapped using the bin mapping approach where a set of six plants (the bin set) permit wselective' or 'bin' mapping using the diploid strawberry mapping population [22]. Only ten new markers were mapped in the T×E population: the eight *Fragaria *ESTs were bin mapped [15] and the two SSRs were mapped using the whole population.

**Table 3:** Anchor markers used for map comparison.

Markers	*Prunus *(T×E)	*Fragaria *(FV×FN)	No. anchor markers
RFLPs	Mapped by Dirlewanger et al. (2004)	Mapped^a^	40
*Fragaria *ESTs	Mapped in the transcript map (GDR)	Bin mapped^a^	13
*Fragaria *ESTs	Bin mapped^a^	Mapped by Sargent et al. (2007)	8
*Prunus *ESTs	Mapped in the transcript map (GDR)	Bin mapped (3)^a ^or mapped (5)^a^	8
Microsatellites	Mapped^a^	Mapped (1) by Sargent et al. (2007) or here (1)^a^	2

Origin of the 71 anchor markers used for the comparison between the genomes of *Prunus *and diploid *Fragaria *using the reference populations (T×E for *Prunus *and FV×FN for *Fragaria*) and mapping strategies used: with the whole population ("mapped") or by bin mapping ("bin mapped").

^a^Results obtained in this paper

The *Fragaria *map, constructed with MapMaker as described in the materials and methods, included 228 markers: 172 of the previous map [18], and 55 new markers studied here in the whole population. Three of the markers mapped in the map by Sargent et al. [18], CFVCT028, CFVCT05 and UFFxa03B05, could not be located using the conditions set for mapping in this work. The seven expected linkage groups (FG1–FG7) were detected, and the total genetic distance covered by the FV×FN map was of 568.8 cM with an average density of 2.5 cM/marker and a ratio of 0.30 Mb/cM. The resulting map shows minor rearrangements in comparison to the original obtained by Sargent et al. [18] most of which occurred in the upper part of the FG2. This map is presented in Additional file 1: 'FV×FN reference map' that is included as supplementary information. The *Prunus *map included a total of 564 markers, the 562 described in [14] plus two SSRs mapped here. The map covers a distance of 511,3 cM with an average density of 0.91 cM/marker and a ratio of 0.59 Mb/cM.

**Figure 1:**
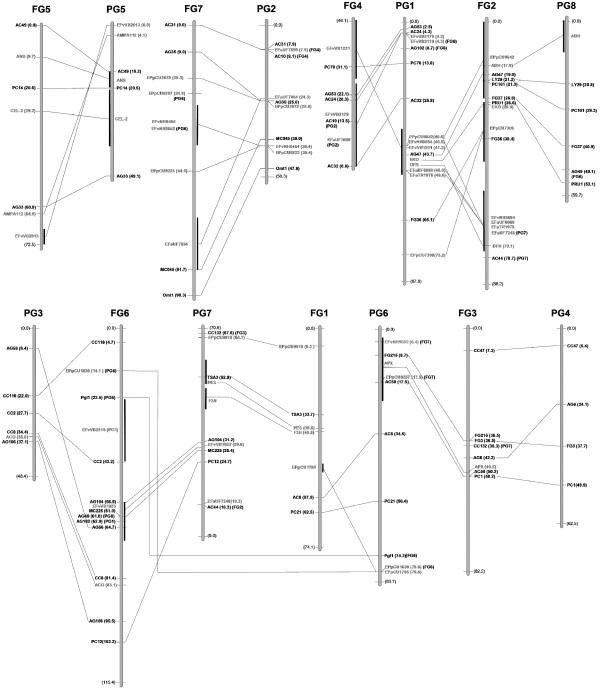
***Prunus-Fragaria *map comparisons**. Comparison between the maps of diploid *Fragaria *(FG1 to FG7) and *Prunus *(PG1 to PG8). Only common markers have been included in the framework of the reference maps of both genera. In parentheses after the markers is the distance from the origin of the linkage group to the marker for markers that have been mapped using the whole *Prunus *or *Fragaria *population. RFLP names are written in black and EST-derived markers in grey. The distance from the origin is not shown for markers that were bin-mapped, which are located within the region of the bin, indicated by a solid vertical bar at the corresponding locations on each of the linkage groups. Markers of one linkage group with correspondence in the other genome to linkage groups other than those that are in the neighbourhood have been indicated by the name of the corresponding group in parenthesis.

The distribution of the 71 anchor markers across the seven linkage groups of the strawberry genome was relatively even (Figure 1), ranging from seven markers on FG1 and FG5 to sixteen markers on FG2, with average marker densities ranging from 10.6 cM/marker on FG1 to 5.5 on FG2. The map distance covered by these markers was 441.3 cM, a 78% of the map constructed with all markers. Only three large gaps of >30 cM were observed, one at the end of FG3 (32.0 cM), one in the middle of FG2 (31.7 cM) and the longest on the lower part of FG7 (37.2 cM). The distribution of these 71 markers across the *Prunus *map was similar, with a maximum of 17 markers on PG1 and a minimum of four on PG4 and marker densities per linkage group ranging from 15.6 (PG4) to 5.0 (PG2) cM/marker. Map coverage with anchor markers was of 399.5 cM, a 78% of the complete map. Longest gaps between markers were smaller in *Prunus*, with only four exceeding 20 cM: 21.7 on PG7; 21.9 on PG6, 20.8 on PG8 and 28.6 on PG5. All the gaps on both the *Fragaria *and *Prunus *maps had at least one marker bin-mapped between the extreme markers of the gap, except for those on PG6 and FG3, suggesting that these were the longest gaps of each map.

### Map comparison between *Prunus *and *Fragaria*

**Figure 2:**
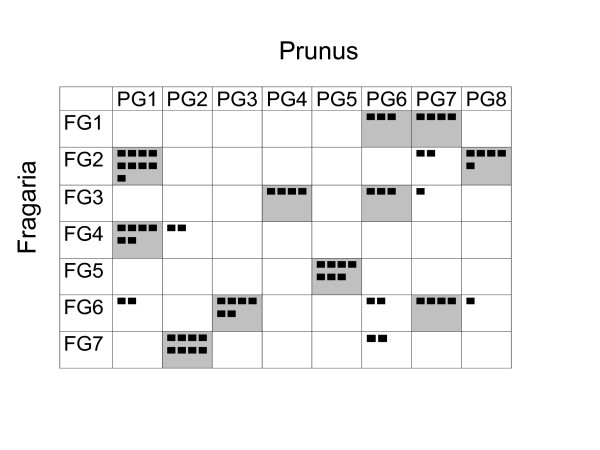
***Prunus-Fragaria *synteny comparison**. Synteny between *Prunus *and *Fragaria*. Number of markers of each linkage group of one genus that correspond to the linkage groups in the other genus. Each marker is indicated by a black dot. Cells that contain three or more markers are noted with a grey background.

The overall pattern of synteny is summarized in Figure 2, where it can be seen that the majority of linkage groups of either species have most markers in one or two linkage groups of the other. The simplest case is G5 of both species which have all markers in common, and the most complex is FG6 which contains markers from five different *Prunus *linkage groups, although most of the markers on this linkage group belong to PG3 and PG7. Considering only the *Prunus *linkage groups, five of them contain most or all markers from only one group of *Fragaria *(PG2-FG7, PG3-FG6, PG4-FG3, PG5-FG5 and PG8-FG2), and the other three of two groups (PG1-FG2 and FG4, PG6-FG1 and FG3, and PG7-FG1 and FG6).

The presence of markers from two or more linkage groups of one species in a linkage group of the other would suggest that a fission/fusion, or a translocation event has taken place between the two species since their divergence from a common ancestor. Nine of these rearrangements would have taken place between the *Fragaria *and the *Prunus *genomes, based on our results.

Colinearity of markers within syntenic regions of *Prunus *and *Fragaria *was only partial. The two most colinear groups FG5 and PG5 require only one inversion event to place all markers in the same order, but many more are required in other linkage groups. As shown in the materials and methods section, FG1 would require two inversions for the markers present on that linkage group to be in the same order as on PG7 and PG6 of *Prunus*, FG3 and FG4, three inversions, FG7 four inversions, FG6 six inversions and FG2 eight inversions. In total, at least 27 inversions are needed to account for the differences in the marker order of the two genomes. The estimated lower boundary of the total number of breakpoints that separate the *Fragaria *and *Prunus *genomes is thus 36 (nine translocations and 27 inversions).

**Figure 3:**
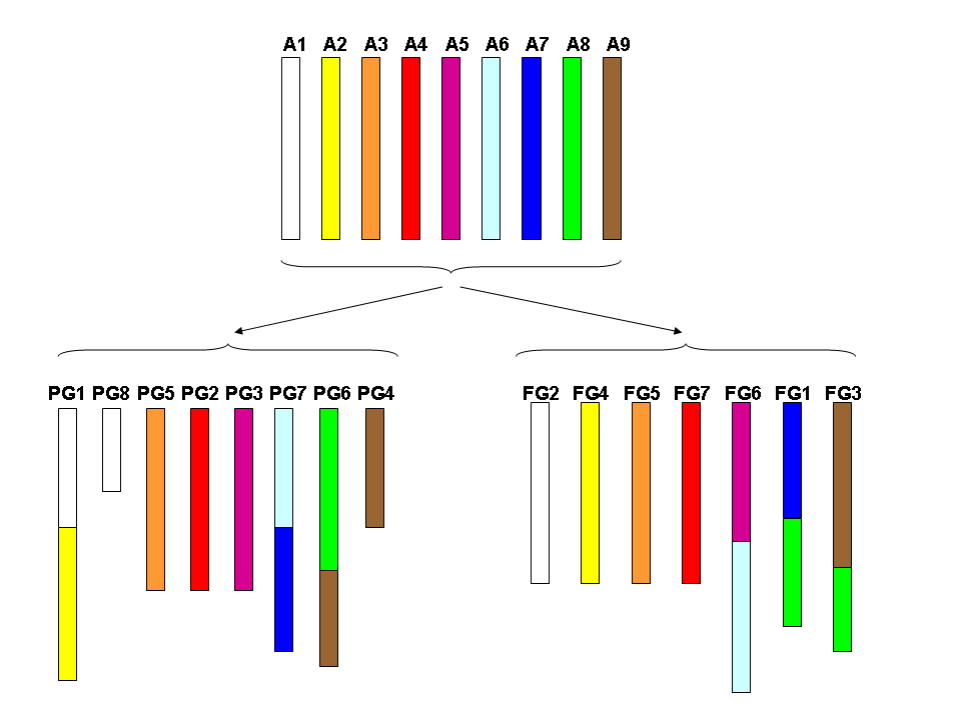
**Scheme of the evolution of *Prunus *and *Fragaria *chromosomes from an ancestral genome**. Model of evolution of *Prunus *(PG1–PG8) and *Fragaria *(FG1–FG7) chromosomes from a hypothetical ancestral Rosaceae genome with *x *= 9 chromosomes (A1–A9). Only major chromosomal rearrangements (fusion/fission or translocation events involving more than two common markers between the two genomes) have been considered.

A scheme of the possible evolution of the *Fragaria *and *Prunus *genomes from a hypothetical ancestral genome of *x *= 9 [23] is presented in Figure 3. In this scheme we have only considered the major chromosomal rearrangements, i.e. fusions/fissions and reciprocal translocations involving more than two of the markers used for the comparison. According to this scenario, the ancestral genome underwent two fusions (A5/A6 and A7/A8, corresponding to FG6 and FG1, respectively) and a reciprocal translocation between part of the A8 chromosome in FG1 and A9, which resulted in FG3, to become the *x *= 7 strawberry genome. For the *Prunus x *= 8 genome, the ancestral genome was submitted to three fusions, A1/A2 to form PG1, A6/A7 to form PG7 and A8/A9 to form PG6 and two fissions, part of A1 in the A1/A2 chromosome to form PG8 and part of A9 in the A8/A9 chromosome to form PG4.

Some inferences about the evolution of certain chromosomes can be formulated based on the comparison between the two maps (Figure 1), the proposed evolution of both genomes from an ancestral one (Figure 3), and the limited information available on cytogenetics and map comparison of the Rosaceae. The simplest comparison is that of FG5 and PG5, which display remarkable levels of structural conservation, the marker order between *Prunus *and *Fragaria *differing by just a single inversion event. Most markers of FG7 and PG2 are in common, suggesting their origin from a single ancestral chromosome (A4), but FG7 includes a short fragment of PG6, and PG2 is not completely included in FG7, with a small region being located on FG4. PG1 is a long linkage group that contains many more markers than the rest and that is likely to coincide with chromosome 1 of *Prunus *[14], which is clearly longer than the other chromosomes of this genus [24]. Based on karyotype observations, such a long chromosome does not exist in *Malus *[25] or in *Fragaria *[26]. The map comparison between *Malus *and *Prunus *[14] provided additional evidence that the long *Prunus *chromosome may be split into two in at least one of the constituent genomes of the amphidiploid *Malus *chromosome complement. Our data suggest that PG1 arose from the fusion of two ancestral chromosomes, A1 and A2, which correspond to FG4 and FG2, respectively, in strawberry. The region of PG1 where the fission/fusion occurred in the *Prunus*-apple comparison (38–44 cM from the top of PG1) is also compatible with that of the *Prunus*-*Fragaria *(estimated to be in the region of 25–45 cM from the top of PG1). Interestingly, most of PG8, one of the *Prunus *chromosomes with the fewest number of markers and with the smallest genetic distance, appears to be integrated into FG2, with five of the six anchor markers studied being in the same order in PG8 and FG2. This situation may be the result of the fission of A1 in *Prunus *but not in *Fragaria*.

## Discussion

The comparison between the maps of *Prunus *and diploid *Fragaria *has been performed with 71 common markers. Most of them were mapped already in the *Prunus *map and were added to the strawberry map here. These markers resulted in good coverage of both genomes: 78% of the total distance of the reference maps of both *Prunus *[14] and strawberry [18]. The average density of anchor markers was of 7.3 cM/marker with a maximum gap of 22 cM (in PG6) for *Prunus *and 8.0 cM/marker and a maximum gap of 32 cM (in FG3) for *Fragaria*. The total map distance of the *Prunus *(519 cM) and the *Fragaria *(569 cM) maps were similar when constructed with all markers available [14,18]. Given that the genome size of *Prunus *is approximately twice that of strawberry these results suggest that the overall recombination rate per physical unit distance for the *Fragaria *FV×FN hybrid was higher than for the almond × peach F_1 _individual that generated the T×E F_2 _population.

For the comparison between the two genomes, we selected 65 RFLP probes, all from *Prunus *or *Malus *species, which produced good hybridization and were single copy in the *Prunus *genome. These probes were studied in strawberry with the same stringency conditions as in *Prunus *and 16 (24%) produced poor or no hybridization, suggesting that they were not present in the strawberry genome or that their sequences differed substantially from those of *Prunus*. Some of the probes that did not hybridize were located together in the same regions of the *Prunus *genome where several probes covering short genetic distances (<10 cM per region) did not hybridize, such as the central region of PG1 and the upper extreme of PG6 (Table 1). The three probes that did not hybridize in the PG1 region were two from a peach cDNA library (PC85 and PC15) and one from a genomic library of *P. ferganensis *(FG16). Two of them (PC15 and FG16) were sequenced (Table 1) and had homology with known proteins. The region of PG6 had four markers (AG13, AG54, AG40 and PLG59), all of them from *Prunus *genomic libraries. Only AG40 was sequenced and its sequence had no homology with protein sequences. These results suggest that the corresponding regions, or at least the specific sequences tested, may be deleted in the strawberry genome. Given that some of the probes are homologous to proteins that are usually present as gene families such as polygalacturonase (FG16) and defensin protein 1 (PC15), an alternative explanation is that they may correspond to copies of these genes with high sequence divergence from those present in strawberry.

From the 40 RFLP probes that produced good hybridization and that were polymorphic, 14 (35%) detected two loci in strawberry, whilst in *Prunus *they were single-locus (Table 1). Tanksley et al. [27] found that five (12%) of the 42 tomato cDNA probes which they mapped in pepper had a different number of copies in each species. These differences in copy number may be due to differential deletions of duplicated DNA fragments existing in the ancestral genome from which *Prunus *and *Fragaria *originate. The presence of these duplications complicates the genome comparison because, if only one locus of the two can be mapped, as with the RFLPs mapped here in *Fragaria*, in half of the cases, the position of the marker could be interpreted as the presence of a spurious genetic rearrangement.

The number of RFLPs mapped in T×E that were single copy, detected with Rosaceae probes, segregated in the strawberry population and had a good distribution along the *Prunus *map, was insufficient for a good coverage of the *Prunus *genome. This was due in part to the fact that approximately half of the probes used for RFLP mapping in *Prunus *have more than one copy [28,29] and that a substantial number of probes used in T×E (30%) come from families other than the Rosaceae [14]. To solve this problem, we used the available EST and physical map information in *Prunus *to find ESTs placed in most of the uncovered regions. We then mapped these ESTs by an efficient and expensive approach, resequencing, using a cheap mapping strategy, bin mapping. This allowed us to cover most gaps of the *Prunus *genome and to reduce the maximum gap without anchor markers in this species to 22 cM. Given the fast rate of growth of the information on the *Prunus *transcript map, this strategy is likely to allow us a more detailed analysis of synteny in the near future.

Figure 2 shows that the level of synteny between *Prunus *and *Fragaria *is high, with most of the markers that mapped to the same linkage group in one species mapping to one or two linkage groups in the other. However, the colinearity is only partial, with an estimated number of nine chromosomal rearrangements involving two chromosomes (translocations or fusion/fission events) and 27 inversions. The number of translocations and fusion/fission events is high if we compare it with other confamilial comparisons where this number has been estimated. Only the distant Solanaceae genomes of pepper and tomato, with 10 of these rearrangements estimated [30], yielded similar results. However, the predominance of inversions over other rearrangements that we found in the *Prunus*-*Fragaria *genome comparison is frequent in the Plant Kingdom, such as in the comparisons between tomato and potato [31], tomato and eggplant [2], tomato and pepper [30], *Brassica nigra *and *Arabidopsis thaliana *[32] or a. *thaliana *and *A. lyrata *[33].

The centromeres or heterochromatic regions around the centromeres are often where breakpoints occur, leading to chromosomal rearrangements. Most inversions and translocations in the Solanaceae [30,34] and Poaceae [35] had their breakpoints at or near the centromere. This information may be useful to deduce the position of the centromeres of some of the *Prunus *or *Fragaria *chromosomes. For example, based on the position where we hypothesize that a fusion event occurred between ancestral chromosomes A1 and A2 to form PG1, we may infer that the centromere is located in the central part of this chromosome (30–45 cM from the top). This is consistent with the metacentric nature of peach chromosome 1. Schubert [36] proposed a model for chromosome fusion where a reciprocal translocation between an acrocentric or telocentric chromosome and another chromosome may generate a larger fused chromosome plus a small chromosome that is eventually lost. Following this model, one of FG2, FG4 or both, or their ancestral chromosomes (A1 and A2), was probably acrocentric. Other metacentric chromosomes may be those that are composed of parts (possibly entire translocated arms) of two chromosomes of the other species; this may be the case of PG7, PG6, FG1, FG6 and FG3 (see Figure 3). In the case of PG6, a reciprocal translocation with PG8 was previously reported in peach [37]. The breakpoint was estimated to be located in the region of 18–39 cM from the top of PG6, which coincides with the region of junction between the fragments of FG1 and FG3 (18–35 cM) and may correspond to centromeric regions of these three chromosomes. On the other hand, the translocation breakpoint of PG8 [37] is located in its distal region, suggesting that it is an acrocentric chromosome.

A rate of 0.14(± 0.06) structural mutations per chromosome per million years (My) of divergence was estimated [6] from the analysis of various macrosynteny comparisons in plants. Considering this rate, our estimation of a number of at least 36 chromosomal rearrangements between *Prunus *and *Fragaria*, assuming that the initial number of chromosomes of the common ancestor was *x *= 9, allows us to estimate that the divergence of these two species dates from approximately 29 Mya. This places these two genera as distant taxa within the Rosaceae, similar to maize and sorghum in the Poaceae (~24 Mya) but closer than tomato and pepper in the Solanaceae (~40 Mya), maize and rice or wheat and rice, both with an estimated divergence time of ~66 Mya [6].

Our results indicate that there is sufficient synteny between the genomes of *Fragaria *and *Prunus *to allow the information on marker or gene or quantitative trait locus (QTL) position from one of these species to be used in the other. For example, the gene that determines the ability to produce runners (vegetative propagules) in strawberry (*R*/*r*) is located on FG2 at a position syntenic to the region of PG1 where the Evergrowing gene (*Evg*/*evg*) that determines continuous leaf production [38] and a QTL that determines blooming time in peach are located [39]. The gene for seasonal vs. perpetual flowering in strawberry (*S*/*s*) maps to a region of FG6 (proximal to the SSR EMFn017), that in our comparison roughly coincides with a PG7 fragment where a major QTL determining blooming time in peach lies [39]. These comparisons are however preliminary and need to be studied in more detail, but are a first insight into other possible comparisons that may facilitate the advancement on the knowledge of the genetics of key characters of the Rosaceae.

## Conclusion

Whilst the economical importance of peach and its relatively easy manipulation (shorter intergeneration period and self-compatibility) compared to other fruit tree species have determined that many genes have been studied [40] and that the position of at least 28 of them has been established on the *Prunus *map [14], the diploid strawberry has important advantages, i.e., a genome of a size similar to that of Arabidopsis, ease of genetic transformation, and a rapid life-cycle. In addition, the plants are small, and produce a large number of seed per cross, and thus diploid strawberry may become a very efficient organism for reverse genetics and other genomics applications that may provide useful information for other Rosaceous species, particularly fruit tree crops [41]. This will be facilitated by the information on map comparisons between these two genera that we present in this paper.

## Methods

### Plant material

The parents and progeny of two mapping populations were used for comparing the *Prunus *and *Fragaria *genomes, those of the F_2_interspecific *Prunus *reference mapping population (N = 82) derived from the F_1 _cross between the almond (*P. dulcis*) cultivar 'Texas' and the peach (*P. persica*) cultivar 'Earlygold' (abbreviated T×E) and the F_2 _interspecific diploid *Fragaria *reference mapping population (N = 76) derived from the F_1 _cross of *F. vesca *815 × *F. nubicola *601 (abbreviated FV×FN). The T×E map is composed of 562 markers (185 SSRs, 361 RFLPs, 11 isoenzymes and 5 STSs) [14], whilst the FV×FN map currently consists of 182 markers (175 SSRs, 6 gene specific markers and 1 SCAR) [18]. Most markers used were selected from T×E and subsequently mapped in FV×FN.

### DNA extraction

For *Fragaria*, one gram of young expanding leaves of each individual was collected and kept at -80°C before DNA isolation. Genomic DNA was isolated from the leaf samples using the CTAB method of Doyle and Doyle [42], followed by DNeasy miniprep kit purification (Qiagen). DNA concentrations were measured using a Gene Quant II spectrophotometer (Pharmacia Biotech). For *Prunus*, DNA was extracted as described in [28].

### RFLP markers

A total of 65 probes from various *Prunus *species and apple were used for RFLP analysis (Table 1). These probes were selected from those used in the construction of the *Prunus *reference map [14] to be single-copy and covering the whole genome at approximately even distances of 10–25 cM. RFLP analysis was performed with the procedure of Viruel et al. [28]. DNA probes were first hybridized in the parents of the FV×FN population and those that detected RFLPs were studied later in all F_2 _individuals.

### Markers based on *Fragaria *or *Prunus *ESTs

An additional set of markers was developed from the *Prunus *transcript map, i.e. the collection of EST unigenes that are located on the same BAC or the same BAC contig of the peach physical map as mapped markers (usually RFLPs) of the reference linkage map [43]. We analysed 515 T×E ESTs currently anchored in the *Prunus *transcript map (found in the Genome Database for Rosaceae) to find homologous EST sequences in *Fragaria *by using the TBLASTX program [44]. The selected sequences (only sequences with an e-value <1.00E-15) (Table 2) were then analyzed with TBLASTN, and the most homologous regions were selected for primer design. Primers were designed to include putative *Fragaria *introns based on the Arabidopsis genome sequence [45] and to give an expected amplicon size >800 bp, using the program Primer3 [46].

For SNP detection, *Fragaria *ESTs were PCR-amplified in the parents of the *Fragaria *mapping population in a temperature-gradient PCR to obtain the optimum annealing temperature for each EST primer pair. The PCR reactions were performed as described in [47] and amplification products were sequenced with an ABI PRISM 3700 DNA Analyzer and SNPs were detected between the two parental lines through sequence alignment using the STADEN package [48] and verified by visual inspection of the DNA chromatograms. The same procedure was followed for *Prunus *ESTs of the transcript map for which we did not find homologous *Fragaria *ESTs.

The sequences of the primers used for the development of these markers can be found in Table 4. The terminology used for the EST derived markers was: E (for EST), a two letter code for the species where the EST was obtained (Fv, Fa or Pp for *F. vesca*, *F. x ananassa *and *P. persica*, respectively), two additional letters for the place where the EST was obtained (i.e. NH for University of New Hampshire, VB for Virginia Bioinformatics Institute, UF for University of Florida, TR for Trisaia Research Center and CU for Clemson University) and four numbers that correspond to the last four digits of the EST EMBL accession number.

**Table 4:** Gene and EST-based marker primer sequences. Primers used for DNA amplification in the markers obtained from *Fragaria *gene or EST or *Prunus *EST sequences.

Origin	Locus name	Forward primer sequence 5' – 3'	Reverse primer sequence 5' – 3'	Reference^a^
*Fragaria *gene	EKO	ACAGTCCAGCTCCAATAGTTCC	GCTTTCCCATTGATTCTTGTCC	AY462247
	F3H	GAGTTGATACCAAGCTCATCTCG	GTCACCTCTCTCCATCCTTCC	AB097151
	DFR	CCACTCCTATGGATTTTGAGTCC	CTAGCACCCCATTTATTGTTGG	AB095030
	ADH	GCKTCAMGAATTATYGGKGTTG	ATGGGASTTKRTGGGTGATG	X15588
*Fragaria *EST	EFvVB2179	ATCTGCGTGACAATGCAAAG	AAGAGCCTTCAGTTGCTCCA	CX662179
	EFvVB2119	GCTCGAGCTGATTACGATTACC	TAAAGGACCCATCAGAGAAACG	CX662119
	EFvNH8894	GGTTAGGTCTCCCCAGTGGT	GGACGTCTCCCACTAGCATC	DV438894
	EFvVB1231	CCAACTGTGACATCCACGAC	GCTGTCACGCAGAAAATCAA	CX661231
	EFaUF6868	GCTCTTCCAGGTCGAGTACG	GTTTCCACTTGGGCAGTTGT	CO816868
	EFaTR1976	GTTGGTGCTGAGTTTGGTGA	CCCAACTGCTCAAGAAGGAG	CO381976
	EFaUF7699	GTTCTTGTTGGTGGAAGCAC	CCTCAAAGACCTGAATGGAG	CO817699
	EFaUF7084	CAGAAGAGGTTCAAGTTCC	ACACCATAGCAAGCCCTG	CO817084
	EFvNH8484	TTCTGGTGTCGGCAAGTC	AGGCCTGCTCAACATTGG	DV438484
	EFvVB2013	GTGCAGTTGCCAAAGGAGC	AGCTGGGTTTGCTGCTT	CX662013
	EFvNH9852	TTCTGTCGTGGTGTCCC	ATGATCTTTTGGCGACCA	DV439852
	EFvNH7822	GATGCTGGGTCTGCTGGG	GCCTGCTCATTGGCATA	CO817822
	EFaUF7248	ACTGCTCGCCCAATGAAG	TCACATCAGCATCCATGTCA	CO817248
	EFavB1923	GGCCGTGTCTCTTGCAGT	TGGGAGCAAATCCACCTT	CX661923
	ACO	AGCACCTTCTACCTCAAACACC	CTCACAGAACAAGTCCAAGAGC	AF129073
*Prunus *EST	EPpCU9642	TTCAGTTGGCAGATCCTGTG	TGCTGAGACCCTTCCAATTT	BU039642
	EPpCU1785	TTTTCCAAACCTTGCTGGAG	GCAGTAGCTGTGGCAATGAA	BU041785
	EPpCU7308	GGCAGGCCGCTCTTATACTA	GACTCTTTTCGGGGTTCCA	BU047308
	EPpCU9257	CACCACCGTTTCAAAAGAGG	CTGAAGCTCTAGCTGAGGCAAG	BU039257
	EPpCU1830	TGATGCAATTGGCACAAAGC	CCTATCACCACTTACTTCACTGC	BU041830
	EPpCU9910	ATAACTCTGCCATCCGAATCC	CATTCCTTGAACAGATCCTTGC	BU039910
	EPpCU9223	AACAGAGCCAAGCTTATGCAG	TTTCTGCGCAACCGCATC	BU039223
	EPpCU2875	AACTCAGAGACATATCTGCACAGG	AAGTTGAAGCGGTCTTCATAGG	BU042875

^a^EMBL accession numbers for the sequences from which novel primer pairs were designed

### *Fragaria *gene-specific markers

Eight further gene specific loci that had previously been mapped in the FV×FN population [21] were mapped in the T×E population. The primer pairs used for amplification of four of these genes (CEL-2, PES, ANS and APX) were previously described by Sargent et al. [21] whilst primer pairs for the remaining four (EKO, F3H, DFR and ADH) were designed following the procedures of Sargent et al. [21] from gene sequences deposited in the EMBL database and are listed in Table 4. The primer pairs were used to amplify products from the parents of the *Prunus *mapping population following the procedure of Sargent et al. [47] and SNPs were detected as described above.

### Microsatellite markers

One SSR (ACO) developed in the 5' UTR of *Prunus *1-aminocyclopropane-1-carboxylate oxidase gene (ACC oxidase; AF129073) was used to locate this gene, which was already mapped in *Fragaria *[21], onto the T×E map. The primers used are listed in Table 4. Another apricot SSR, AMPA112, previously described by Hagen et al. [49], was polymorphic in both reference populations and was also included in the map comparison. PCR reactions were performed as described by [50].

### Map construction in *Fragaria *and bin mapping in *Prunus *and *Fragaria*

The T×E map of Dirlewanger et al. [14], constructed with MapMaker/EXP v. 3.0 [51], was used as a standard for map comparisons. Data for the FV×FN map, originally constructed with Joinmap 3.0 [52] by Sargent et al. [18], was used for the mapping of novel loci in *Fragaria*. However, in order to make both maps comparable, the same data of the FV×FN map plus those obtained here were used, but the map was reconstructed with MapMaker. The Kosambi mapping function was used to convert recombination units into genetic distances. The mapping procedure followed the guidelines of previous maps constructed in *Prunus *[29]. The usual notation for the eight linkage groups of *Prunus *is G1 to G8 and for the seven *Fragaria *groups is I-VII. In order to facilitate the map comparison we have used on this occasion the terminology PG1–PG8 for *Prunus *and FG1–FG7 for *Fragaria*.

A subset of six plants of the T×E mapping population having a high number of recombination breakpoints and a uniform distribution across the *Prunus *genome was selected by Howad et al. [15]. The genotype of this set of plants (the bin set) identified 64 fragments (bins) of the *Prunus *map with average size 7.8 cM. Following a similar approach, a bin set of six plants was selected from the FV×FN mapping population by Sargent et al. [22]. The *Fragaria *bin set detected 46 fragments of its genome with an average length of 12.6 cM. Using the bin sets of both species allowed us to establish the position of some of the markers with lower cost and effort than mapping with the whole population.

### Mapping strategy

Table 3 summarizes the mapping strategy employed for all novel markers mapped in this investigation. All RFLPs used were already mapped in *Prunus *using the entire T×E progeny and were mapped in *Fragaria *using the entire FV×FN population. The *Fragaria *and *Prunus *ESTs found to be polymorphic in the parents of the *Fragaria *mapping population were either sequenced in the *Fragaria *bin set and bin-mapped, or mapped in the whole FV×FN population when clear intron length polymorphisms were detected. The eight *Fragaria *ESTs, previously mapped in FV×FN, were bin mapped in *Prunus*. The SSRs were mapped in *Prunus *and *Fragaria *using all individuals of these populations. The data for each marker were scored independently by two researchers. Conflicting results were re-examined and in case of disagreement, the most conservative option was taken.

### Estimating the number of chromosomal rearrangements

To estimate the number of chromosomal rearrangements that have occurred between *Prunus *and *Fragaria *since they diverged from a common ancestor, we elaborated a list of the markers of each of the *Fragaria *linkage groups with their correlative position on the *Prunus *linkage map. Taking FG1 as an example, this linkage group has seven anchor markers with order 7.2 (PG7, position 2), 7.3, 7.5, 7.4, 6.10, 6.6, 6.7. Then, we deduced the minimal number of mutations that would place the positions of these markers in the same order as in *Prunus*. In the example of FG1, we counted one translocation (between PG7 and PG6) and two inversions, one involving the 7.4–7.5 fragment and the other the 6.6–6.7 fragment, to give the final marker order: 7.2, 7.3, 7.4, 7.5, 6.10, 6.7, and 6.6. An additional mutation (probably caused by a translocation) had to occur to explain the gap between 6.10 and 6.7. This mutation was considered when analyzing the chromosome that received the translocated fragment. We counted one breakpoint per translocation and one per inversion. This is a lower boundary, as inversions may require one breakpoint if they involve the distal part of a chromosome, or two if they correspond to an internal fragment of a chromosome. Finally, we considered as translocations only those regions comprising two or more markers, as single-locus translocations are more likely to be spurious.

## Authors' contributions

AM and PA conceived and designed the experiments. SV carried out the experiments with RFLPs in the *Fragaria *mapping population, DJS performed experimental work for mapping gene-specific markers in the *Fragaria *and *Prunus *bin mapping populations, and AM carried out the experimental work for mapping EST in the *Fragaria *population. PA coordinated the preparation of the manuscript to which all authors contributed, and read and approved the final manuscript.

## Supplementary Material

Additional file 1 **FV×FN reference map**. The diploid *Fragaria *reference map constructed using MapMaker from the data of Sargent et al. (2006) and the novel markers (boldface and red) added in this paper. Click here for file
