# Supplementary figures and images for: Synteny conservation between two distantly-related Rosaceae genomes: Prunus (the stone fruits) and Fragaria (the strawberry)
Source: BMC Plant Biol. 2008 Jun 18;8:67. doi: 10.1186/1471-2229-8-67 (PMC2442709; doi:10.1186/1471-2229-8-67)

## FG1

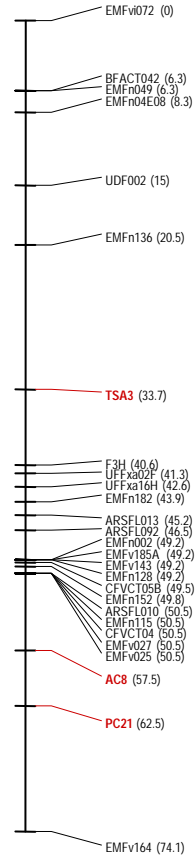

## FG2

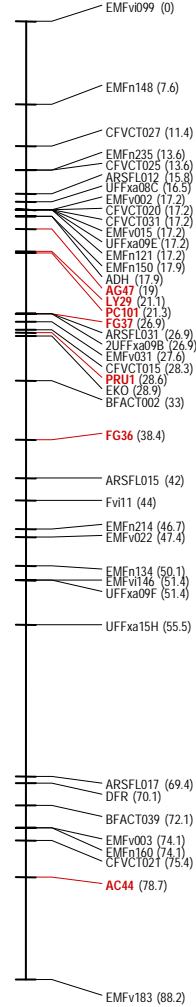

## FG3

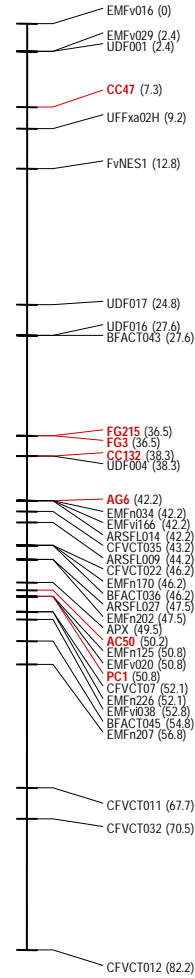

## FG4

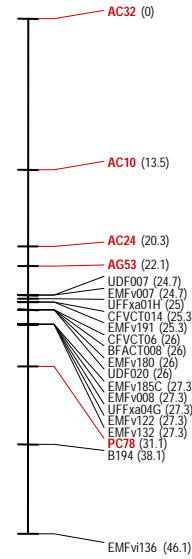

## FG5

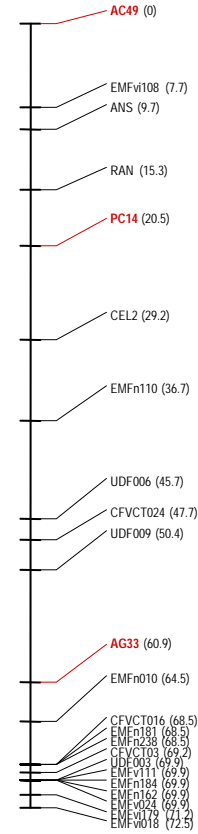

## FG6

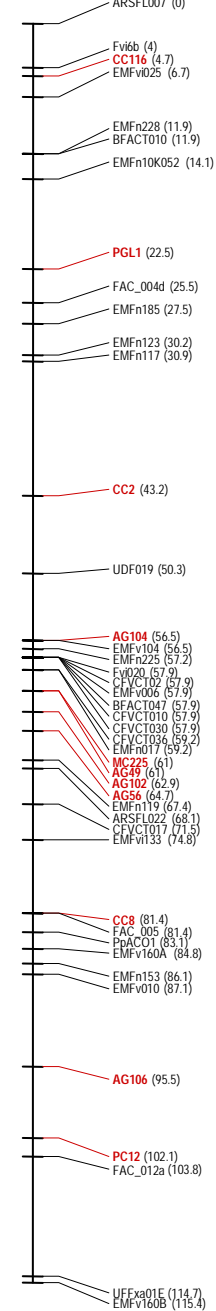

## FG7

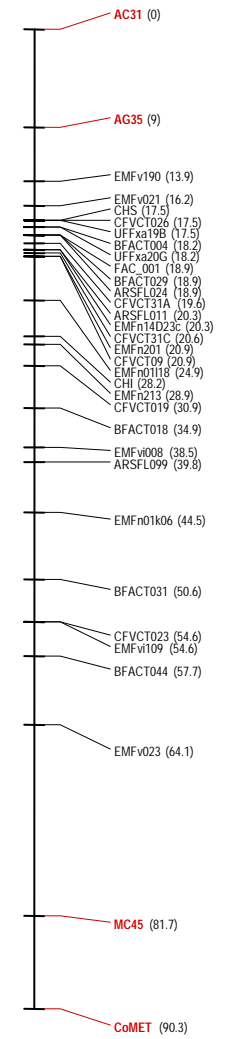

Supplement: Additional file 1 — FV×FN reference map. The diploid Fragaria reference map constructed using MapMaker from the data of Sargent et al. (2006) and the novel markers (boldface and red) added in this paper. [file 1471-2229-8-67-S1.pdf]
